# Prevalence and genotype distribution of human papillomavirus (HPV) among adolescent girls and young women in a high HIV burden rural area of South Africa: a cross-sectional survey

**DOI:** 10.64898/2026.09.09.26362609

**Published:** 2026-09-11

**Authors:** Maphe Mthembu, Kathy Baisley, Jacob Busang, Nqobile Ngoma, Nonsikelelo Ndlela, Nonhlanhla Okesola, Carina Herbst, Thandeka Khoza, Jaco Dreyer, Lenine Liebenberg, Zaza Ndhlovu, Maryam Shahmanesh

## Abstract

**Background:**

Human papillomavirus (HPV) is the primary cause of cervical cancer, with women living with HIV at increased risk of HPV infection and HPV-related disease. Data on HPV prevalence and genotype distribution among adolescent girls and young women (AGYW) in rural South Africa settings remain limited. We estimated HPV prevalence, genotype distribution, and factor associated with HPV infection among AGYW in a high HIV prevalence district of KwaZulu-Natal, South Africa.

**Methods:**

This cross-sectional study was nested within the baseline survey of the Thetha Nami population-based trial among young people aged 15-30 years in rural uMkhanyakude District, KwaZulu-Natal. Survey participants were asked to provide dried blood spots for HIV testing and genital swabs for STI testing. For this study, we selected AGYW living with HIV with available vaginal swabs and a frequency-matched sample of AGYW without HIV. HPV genotyping was done using the HPV Direct Flow Chip Kit (Master Diagnostica, Spain). Genotype-specific HPV prevalence was described overall and by HIV status. Logistic regression was used to identify factors associated with HPV infection.

**Results:**

Among 187 AGYW with valid HPV results, 90 (48.1%) were living with HIV. Overall HPV prevalence was 69.1% (95% confidence interval (CI)=54.5-80.7%) and high-risk (hr)HPV prevalence was 57.3% (95%CI=44.1-69.6%). HPV prevalence was higher among AGYW living with HIV than among AGYW without HIV (84.6% vs 64.6%, p=0.02). HPV genotype distribution varied by HIV status: HPV35, HPV45 and HPV58 were the most prevalent hrHPV genotypes among AGYW living with HIV, vs HPV52, HPV56 and HPV31 among AGYW without HIV. No HPV16 or HPV18 infections were detected among participants who were age-eligible for the national HPV vaccination programme. Reported condomless sex (adjusted odds ratio (aOR)=2.23, 95%CI=1.06–4.70) and the presence of another sexually transmitted infection (aOR=4.30, 95%CI=1.52–12.20) were associated with HPV infection.

**Conclusions:**

AGYW in rural uMkhanyakude experience a high burden of hrHPV infection, especially those living with HIV. Regardless of HPV vaccination status not being confirmed, no HPV 16/18 was detected among vaccine-eligible AGYW. The higher prevalence of non-HPV16/18 hrHPV genotypes covered by the nonavalent vaccine highlights the potential value of vaccines with broader genotype coverage. These findings support expanding HPV screening and cervical cancer prevention programmes in high HIV prevalence settings.

## Full Text Availability

The license terms selected by the author(s) for this preprint version do not permit archiving in PMC. The full text is available from the preprint server.

